# Nitric oxide synthase expression correlates with death in an experimental mouse model of dengue with CNS involvement

**DOI:** 10.1186/1743-422X-10-267

**Published:** 2013-08-26

**Authors:** Kátia Paulino Ribeiro de Souza, Emanuele Guimarães Silva, Eliseu Soares de Oliveira Rocha, Leandra Barcelos Figueiredo, Camila Megale de Almeida-Leite, Rosa Maria Esteves Arantes, Juliana de Assis Silva Gomes, Gustavo Portela Ferreira, Jaquelline Germano de Oliveira, Erna Geessien Kroon, Marco Antônio Campos

**Affiliations:** 1 Universidade Federal de Minas Gerais, Belo Horizonte, Minas Gerais, Brazil; 2 Universidade Federal do Piauí, Parnaíba, Piauí, Brazil; 3 Centro de Pesquisas René Rachou, Fundação Oswaldo Cruz, Belo Horizonte, Minas Gerais, Brazil

**Keywords:** *Dengue virus*, Murine model, Neurovirulence, Neuropathogenesis, Immunopathogenesis, Encephalitis, Nitric oxide synthase 2, Nitric oxide, Interferon gamma

## Abstract

**Background:**

The clinical presentation of dengue is classified by the World Health Organization into dengue without warning signs, dengue with warning signs and severe dengue. Reports of neurological disease caused by *Dengue virus* (DENV) are becoming frequent, with symptoms that include reduced consciousness, severe headache, neck stiffness, focal neurological signs, tense fontanelle and convulsions. However, the immune mechanisms involved in neurovirulence remain poorly understood. Here we present a mouse model in which one genotype of DENV is inoculated by the intracranial route and infects C57/BL6 mice and replicates in the brain, causing death of mice.

**Methods:**

Mice were infected with different serotypes/genotypes of DENV by the intracranial route to evaluate viral replication, host cytokine and nitric oxide synthase 2 (*Nos2*) expression in the brain via real-time PCR. Histological analysis of the brain tissues was also performed. An analysis of which cells were responsible for the expression of cytokines and *Nos2* was performed using flow cytometry. Survival curves of infected animals were also generated

**Results:**

DENV 3 genotype I infected mice and replicated in the brain, causing death in our murine model. The increased levels of NOS2 could be the cause of the death of infected mice, as viral replication correlates with increased *Nos2* and cytokine expression in the brain of C57BL/6 mice. In *Nos2*^−/−^ mice that were infected with DENV, no clinical signs of infection were observed and cytokines were expressed at low levels, with the exception of interferon gamma (*Ifng*). Additionally, the *Ifng*^−/−^ mice infected with DENV exhibited a severe and lethal disease, similar to the disease observed in C57BL/6 mice, while the DENV- infected *Nos2*^−/−^ mice did not display increased mortality. Analyses of the brains from infected C57BL/6 mice revealed neuronal degeneration and necrosis during histopathologic examination. IFNg and NOS2 were produced in the brains of infected mice by CD4^+^ T cells and macrophages, respectively.

**Conclusion:**

The neurovirulence of DENV 3 genotype I is associated with a deleterious role of NOS2 in the brain, confirming this murine model as an appropriate tool to study DENV neurovirulence.

## Background

The clinical signs of neurological disease caused by *Dengue virus* (DENV) are reduced consciousness, severe headache, neck stiffness, focal neurological signs, tense fontanelle, and convulsions [1]. The pathophysiology of neurological involvement in dengue infection is attributed to several factors, including cerebral edema, cerebral hemorrhage, cerebral anoxia, microcapillary hemorrhage, and the release of toxic products [2]*.* Therefore, dengue infection is considered to be a cause of encephalitis and other neurological manifestations in endemic regions [1,3-12]*.* Studies have shown that DENV can interact with various cell types including dendritic cells, monocytes, macrophages, hepatocytes and endothelial cells [13]*,* resulting in the production of immune mediators that are present during severe DENV infection. High levels of cytokines, such as TNF alpha (TNFa), IFN gamma (IFNg) [14,15], have been detected in patients with severe dengue. However, it is still not clear how these cytokines are induced or what these cytokines’ role is in dengue pathogenesis. Despite many *in vivo* and *in vitro* studies that have attempted to determine the role of various cytokines [9,13,16-18], the lack of small animal models that simulate dengue human symptoms limits the dissection of the mechanisms of dengue pathogenesis [11].

Previous work in our laboratory identified DENV-3 genotype I in serum samples from patients in Minas Gerais classified as having severe dengue; subsequently, the same genotype was found in naturally infected field-caught *Aedes aegypti* mosquitoes and eggs [19,20]. We previously reported the virulence of low-passage isolates of DENV-3 genotypes I and III isolated from Brazil in C57BL/6 mice inoculated by the intracranial (i.c.) route. We observed that the DENV-3 genotype I isolate caused neurological disease, whereas infection with the DENV-3 genotype III isolate was asymptomatic [21].

To better characterize and understand the immunopathology and neurovirulence that occurs in dengue infected hosts, we used DENV-3 genotype I isolates obtained from fatal human dengue cases (here named MG20 and MG21) to experimentally infect mice in this study, causing a dose-dependent fatal neurological disease. We also demonstrated that after i.c. inoculation, the virus isolate MG20 DENV-3 genotype I induced higher levels of expression of cytokines and pro-inflammatory mediators including nitric oxide synthase (NOS2) in the brain. We showed that this virus had reduced neurovirulence in *Nos2*^−/−^ mice. The mortality of *Nos2*^−/−^ mice infected with this dengue isolates was reduced to zero, in contrast with the mortality of the immunocompetent C57BL/6 mice and interferon gamma (*Ifng)*^−/−^ mice, which were 80% and 100%, respectively. These results indicated a correlation between immunopathology and *Nos2* expression in the brain when infection with a neurovirulent virus occurs. Therefore, this murine model can be used as a tool to study dengue neurovirulence.

## Methods

### Virus

*Dengue virus* (DENV-1, BH4); DENV-2 (Pi59); DENV-3 (MG20); DENV-3 (MG21) and DENV-3 (Pi76) isolates from the sera of dengue patients were obtained from the collection of the Laboratório de Virus, UFMG and passaged no more than 6 times in C6/36 cells. DENV-1 Mochizuki [22] was kindly provided by Prof. Luiz Tadeu Figueiredo, USP, SP, Brazil, and DENV-4 (Boa Vista, 1982) [23] was provided by Prof. Mauricio Lacerda Nogueira, FAMERP, USP, SP, Brazil. Viral stocks were generated in C6/36 cells infected at a multiplicity of infection (moi) of 0.01. To produce viral stocks, the supernatant was harvested, cell debris was removed by centrifugation at 2,000 × g for 5 min, and the viral supernatant was stored at −70°C.

### Cells

C6/36 cells (American Type Culture Collection, Manassas, VA) were maintained in Leibowitz (L-15) medium (Gibco, USA) supplemented with 5% heat-inactivated fetal bovine serum (Cultilab, Brazil) and antibiotics in an incubator at 28°C. These cells were used to support virus replication.

BHK-21 cells (American Type Culture Collection, Manassas, VA) were maintained in minimal essential medium (Gibco, USA) supplemented with 5% heat-inactivated fetal bovine serum (Cultilab, Brazil) and antibiotics in 5% CO_2_ at 37°C. These cells were used for virus titration.

### Mice

All of the animal experiments were approved based on the regulations and guidelines of the Ethical and Animal Use Committee on Animal Experimentation (CETEA/UFMG 026/2011).

C57BL/6 (WT) mice were obtained from Centro de Bioterismo–Universidade Federal de Minas Gerais (MG, Brazil). The *Nos2*^−/−^ and *Ifng*^−/−^ mice on the C57BL/6 background were obtained from Centro de Criação de Animais de Laboratório–Fundação Oswaldo Cruz (RJ, Brazil). Male mice at 8–10 weeks of age were inoculated via the intracranial route with 20 μL of the virus or uninfected C6/36 cell supernatants as a control. The mice were anesthetized using ketamine (Agribrands do Brasil Ltda, Brazil).

### Plaque reduction neutralization test (PRNT_50_)

The plaque assay in BHK-21 cells was performed using a protocol that was modified from Russell and others (1967) [24]. Briefly, BHK-21 cells were seeded into 24-well plates at a density of 1 × 10^5^ cells/well and incubated overnight at 37°C in 5% CO_2_ until the cells were approximately 80% to 90% confluent. Serum samples were inactivated at 56°C for 30 min and serially diluted in 2-fold steps (1:20 to 1:120), and the virus (containing 40–60 pfu of DENV-3) was preincubated with the sera in a final volume of 360 μL for 1 h at room temperature. The cell monolayer was washed with PBS, and 150 μL of the virus and serum mixture was added to the cells for 1 h at 37°C and overlaid with 1 ml per well of MEM containing 1.5% FBS, 1.5% carboxymethyl cellulose (Sigma-Aldrich, USA) and antibiotics. The plates were incubated for 5 days at 37°C in 5% CO_2_. Controls were included, and the test was performed in duplicate. The cells were fixed with 4% formalin for 1 hour and stained with 0.8% crystal violet (Sigma-Aldrich, USA) for 15 min. The plaque counts were averaged and are presented in Units per ml, which corresponds to the reciprocal of the dilution that neutralized 50 percent of the plates in 1 ml serum.

### RNA extraction

The brains were aseptically removed from mice and stored at −70°C until processing. RNA extraction was performed using the TRIzol reagent (Invitrogen) according to the manufacturer’s instructions. The extracted RNA was quantified with a Nanodrop ND-1000 spectrophotometer (Thermo Scientific) at 260 and 280 nm.

### Reverse transcription

Reverse transcription was performed according to the procedures provided by the manufacturer of the M-MLV RT enzyme (Promega, Madison, WI) using 3 μg of RNA.

### Real-time PCR

Real-time PCR [25] was performed to measure the mRNA expression levels of cytokines, chemokines and *Nos2* and to measure the 5’UTR genomic region of DENV in the indicated mice. The reactions were performed using the SYBR Green PCR Master Mix (Applied Biosystems, Carlsbad, CA) in a Step One real-time PCR System and the reaction conditions were 50°C for 2 minutes, 95°C for 10 minutes, and 40 cycles of 95°C for 15 minutes and 60°C for 1 minute, followed by a final dissociation stage. The following oligonucleotides were used in the reactions: Hypoxanthine-guanine phosphoribosyl transferase (*Hprt)* (forward: 5’-GTTGGATACAGGCCAGACTTTGTTG-3’; reverse: 5’-GATTCAACTTGCGCTCATCTTAGGC-3’); *Ifnb* (forward: 5’-CTGGAGCAGCTGAATGGAAA-3’; reverse: 5’-GTCTGCTGGTGGAGTTCAT-3’); *Cxcl10* (forward: 5’-GCCGTCATTTTCTGCCTCAT-3’; reverse: 5’-GCTTCCCTATGGCCCTCATT-3’); *Ccl2* (forward: 5’-CTTCTGGGCCTGCTGTTCA-3’; reverse: 5’-CCAGCCTACTCATTGGGATCA-3’); *Ccl3* (forward: 5’-ACTGCCTGCTGCTTCTCCTA-3’; reverse: 5’-TTGGAGTCAGCGCAGATCTG-3’); *Il1b* (forward: 5’-CGCAGCAGCACATCAACAAGAGC-3’; reverse: 5’-TGTCCTCATCCTGGAAGGTCCACG-3’); *Ifng* (forward: 5’- TCAAGTGGCATAGATGTGGAAGAA-3’; reverse: 5’- TGGCTCTGCAGGATTTTCATG-3’); *Tnfa* (forward: 5’- CATCTTCTCAAAATTCGAGTGACAA-3’; reverse: 5’- TGGGAGTAGACAAGGTACAACCC-3’); *Ccl5* (forward: 5’- GCAAGTGCTCCAATCTTGCA-3’; reverse: 5’- CTTCTCTGGGTTGGCACACA-3’); *Nos2* (forward: 5’- CAGCTGGGCTGTACAAACCTT-3’; reverse: 5’- CATTGGAAGTGAAGCGTTTCG-3’); and 5’UTR region of DENV (forward: 5’- TCGGAAGCTTGCTTAACGTAG-3’; and reverse: 5’- TCCGTTGGTTGTTCATCAGA-3’). The relative quantification methodology was used to analyze the data. Gene or viral RNA expression was normalized to the expression level of the constitutively expressed gene *Hprt*. All reactions were replicated.

### Cell preparation and flow cytometry

On the 8th day post infection (d.p.i.), mice were anesthetized and perfused with phosphate-buffered saline (PBS). Brains were removed and the adherent leukocytes were isolated using a previously described protocol [26]. Aliquots of these leukocytes were incubated with APC-labeled anti-mouse F4/80 (eBioscience, USA) to identify macrophages, FITC-labeled anti-mouse NK1.1 (BD Pharmingen, USA) to identify NK cells, APC-labeled anti-mouse CD4 (eBioscience, USA) to identify helper T cells, and PECy5-labeled anti-mouse CD8 (BD Pharmingen, USA) to identify cytotoxic T cells. The cells were washed and fixed with FACS FIX Solution (BD Pharmingen, USA). After fixation the leukocytes were washed twice and submitted to permeabilization by the addition of 150 μL of Perm buffer (FACS buffer supplemented with 0.5% saponin, Sigma-Aldrich, USA) for 10 min. at room temperature. The leukocytes were then incubated with 20 μL of PE-labeled anti-IFNg and FITC-labeled anti-NOS2 (BD Pharmingen, USA) for 15 minutes at 4°C in the dark. After two wash steps, the cells were fixed with FACS FIX Solution and stored at 4°C for cytometric acquisition. One hundred thousand events were acquired for each sample. A FACS Calibur (Becton Dickinson, CA, USA) with CellQuest™ software was used for acquisition and the data were analyzed using the FlowJo 7.2.5 software (Tree Star, Inc., Ashland, OR) and are presented as the percentage of positive cells within the gated population.

### Histological analysis

Brain fragments were fixed in 10% neutral-buffered formalin and processed for paraffin embedding. The brain sagittal sections (4 μm) were stained with hematoxylin and eosin (H&E). The images were acquired using an Olympus BX51 microscope and the Image-Pro Express 4.0 (Media Cybernetics, MD, USA) software.

### Morphometric analysis

Twelve images per specimen were obtained, and perivascular inflammatory areas, which are areas of increased cellularity in the parenchyma immediately close to a small vessel, were manually measured using the Image J 1.45S software (NIH, USA), and expressed as the inflammatory area per 100 μm^2^ of brain tissue. Data from the different groups were compared with one-way analysis of variance (ANOVA) (GraphPad Prism software; San Diego, USA). Statistical significance was set at P < 0.05 when comparing the data from the 8th d.p.i. in C57BL/6 mice with all of the other bars.

### Statistical analysis

Real-time PCR was statistically analyzed using the Kruskal-Wallis nonparametric tests and Dunn’s multiple comparison tests. Kaplan-Meier survival curves were used to display the survival data, and log rank analyses were employed to determine the statistical significance between the experimental groups. To analyze flow cytometry results, nonparametric two-way ANOVA tests and the Bonferroni post-hoc test were performed. The analyses were conducted using GraphPad Prism 5 software for Windows (GraphPad Software, Inc., La Jolla, CA).

## Results

### Nitric oxide synthase expression correlates with death in a mouse experimental model of dengue with CNS involvement

**Figure 1:**
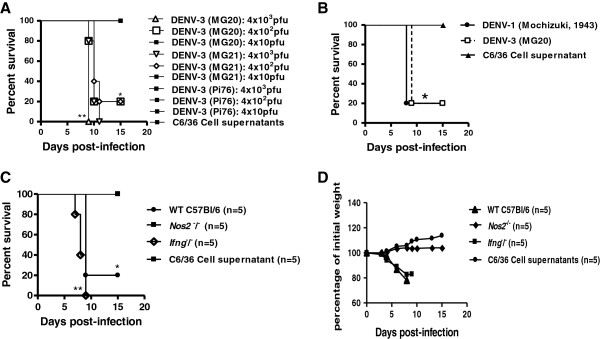
**The genotype I of DENV-3 (MG20) isolate is neurovirulent. ***Nos2*^*−/− *^ mice are resistant to infection, while *Ifng*^*−/−*^ and WT mice are susceptible to infection*.* Mice were intracranially (i.c.) inoculated with DENV and monitored daily until 15 d.p.i. Mice inoculated with uninfected C6/36 cell supernatants were used as controls (n = 5 for all the groups). **(A)** 4 × 10^3^ to 4 × 10 pfu of DENV-3 (MG20, Pi76 or MG21). * P *<* 0.05, ** P *<* 0.01 when comparing 4 × 10^2^ DENV-3 (MG20) and 4 × 10^2^ DENV-3 (MG21) or 4 × 10^3^ DENV-3 (MG20) and 4 × 10^3^ DENV-3 (MG21) with 4 × 10 DENV-3 (MG20), 4 × 10 DENV-3 (MG21) or the three different doses used in the DENV-3 (Pi76), or the cell supernatant. **(B)** 4 × 10^2^ pfu of DENV-3 (MG20) or DENV-1 (Hotta, 1951). *P <0.05, when comparing DENV-1 or DENV-3 (MG20)-infected animals to the supernatant-inoculated animals. **(C)** 4 × 10^2^ pfu of DENV-3 (MG20) in C57BL/6 (WT), *Ifng*^−/−^ and *Nos2*^−/−^ mice. *P *<* 0.05 and P < 0.001, when comparing *Nos2*^−/−^ mice with WT mice or with *Ifng*^−/−^ mice **(D)** Body weight was expressed as a percentage of the initial weight of the animal. Survival curves were statistically analyzed using the Kaplan-Meier method followed by log-rank tests. The results are representative of two similar and independent experiments.

C57BL/6 (WT) mice were i.c. inoculated with concentrations from 4 × 10 to 4 × 10^3^ pfu of low-passage isolates of DENV-1 (BH4); DENV-2 (Pi59); DENV-4 (Boa Vista, 1982), DENV-3 genotype I (MG20), DENV-3 genotype I (MG21), or DENV-3 genotype III (Pi76). The mice were monitored daily to determine their susceptibility to infection and to observe clinical signs of infection such as weight loss, lethargy, ruffled fur and a hunched posture. Inoculation of mice with uninfected C6/36 cell supernatants were used as controls. Only DENV-3 genotype I (MG20 and MG21)-infected mice exhibited clinical signs and mortality when doses of 4 × 10^3^ pfu (100% mortality) or 4 × 10^2^ pfu (80% mortality) were used (Figure 1A). After infection, the mice gradually developed anorexia, asthenia and lost weight. From the 6th to the 8th day after infection (d.p.i.), 80% of the mice that were infected with 4 × 10^2^ pfu of DENV-3 genotype I presented ruffled fur, hunched postures and loss of balance followed by paralysis. Most of these mice died 12–24 hours after the onset of paralysis, which was approximately the 10th d.p.i. Mice that were infected with DENV serotypes 1, 2, or 4, with DENV-3 genotype III, or the C6/36 cell supernatant showed no clinical signs or mortality (Figure 1A). The results demonstrate a significant difference (P < 0.05) between the survival curves of mice after infection with 4 × 10^2^ pfu of DENV-3 genotype I (MG20 or MG21) and the survival curves representing the mice infected other DENV types. In mice infected with lethal doses of DENV-3 genotype I, a clear correlation between the viral dose and the time of death was observed. Mice infected with 4 × 10^2^ pfu of DENV-1 Mochizuki, a neurovirulent mouse-adapted strain isolated in 1943 in Japan [22], presented neurological clinical signs and mortality that were similar to those of mice infected with 4 × 10^2^ pfu of DENV-3 genotype I (Figure 1B). Nitric oxide (NO) is synthesized by macrophages after induction of NOS2 by IFN gamma [27,28], and NO has antiviral activities against encephalitic viruses [29]. Thus, *Nos2*^–/–^ and *Ifng*^–/–^ mice, both on a C57BL/6 background, were also infected with 4 × 10^2^ pfu of DENV-3 genotype I (MG20) and observed daily for the above cited clinical signs of encephalitis and mortality (Figure 1C). Infected WT, *Ifng*^−/−^ and *Nos2*^−/−^ mice were monitored for weight loss over a period of 15 days (Figure 1D). Infected WT and *Ifng*^−/−^ mice presented clinical signs of infection and mortality. WT and *Ifng*^−/−^ mice, which were similar to WT mice, exhibited weight loss starting on the 5^th^ d.p.i. (Figure 1D), progressing to lethargy, ruffled fur and hunched postures with posterior paw paralysis and death between the 7^th^ and 10^th^ d.p.i. (Figure 1C). Only 20% of the infected WT mice survived. Surprisingly, 100% of the *Nos2*^−/−^ mice survived infection with DENV-3 genotype I (Figure 1C) without exhibiting any clinical signs of infection or weight loss until the 15th d.p.i. (Figure 1D). There were significant differences in the mortality rates between the infected WT and *Nos2*^−/−^ mice (P < 0.05), suggesting a pathological role for NO in DENV-3 genotype I-induced neurological disease. To prove that the infection was successful in the infected *Nos2*^−/−^ mice, a plaque reduction neutralization test (PRNT_50_) was performed using sera from the *Nos2*^−/−^ mice at the 30th d.p.i. The titers obtained in this assay were greater than 100 U/ml.

### DENV-3 genotype I (MG20) replicates in the brain, inducing increases in *Nos2* and pro-inflammatory cytokine expression levels

**Figure 2:**
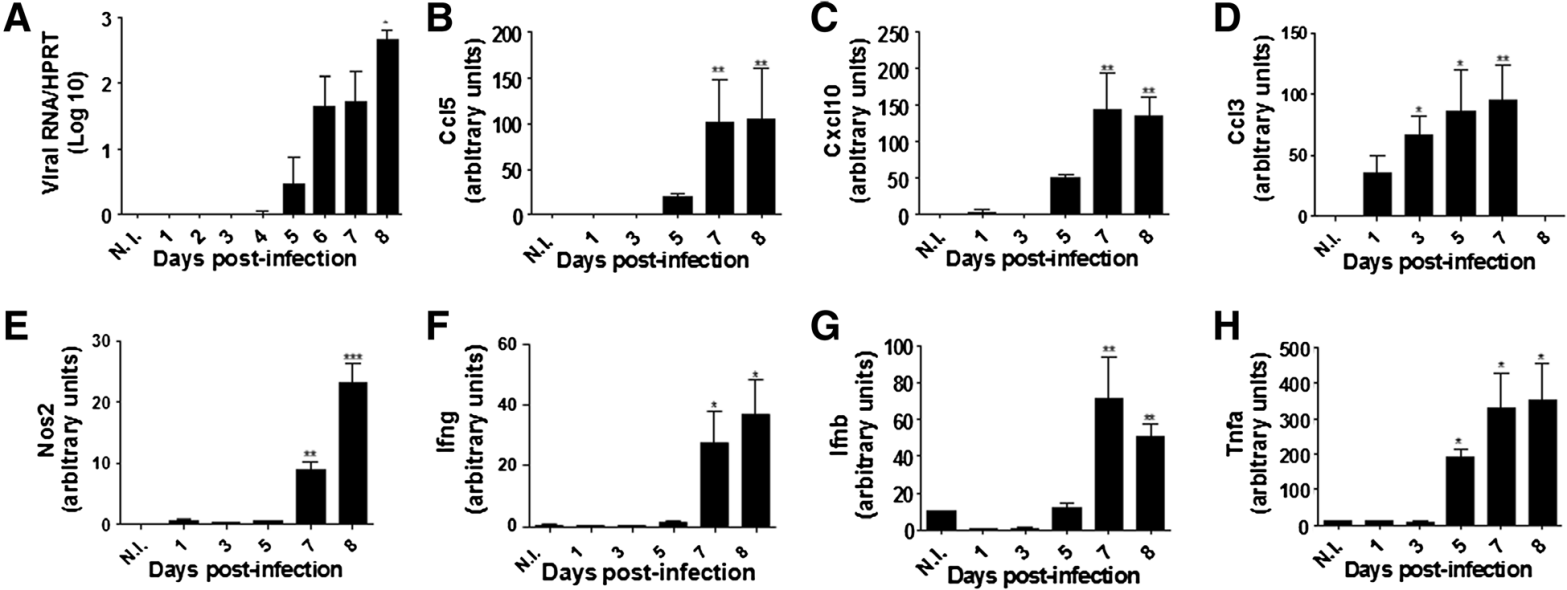
**Immunological profile of WT mice infected with DENV-3 (MG20).** Mice were i.c. inoculated with 4 × 10^2^ pfu of DENV-3 (MG20) and the brains of 4 animals per group were collected each day until the 8th d.p.i. Mice inoculated with uninfected C6/36 cell supernatants were used as controls. RNA was extracted from the brains and reverse transcription and quantitative PCR were performed. **(A)** viral RNA **(B)***Ccl3*; **(C)***Cxcl10*; **(D)***Ccl3*; **(E)***Nos2*; **(F)***Ifng*; **(G)***Ifnb*; **(H)***Tnfa*. *P < 0.05, **P < 0.001 or ***P < 0.0001 when the infected mice were compared with the uninfected control mice (N.I.)*.* The results are representative of two similar and independent experiments. Statistical analyses were performed using Kruskal-Wallis nonparametric tests and Dunn’s multiple comparison tests. Bars represent the SEM.

Four WT infected mice per group were euthanized each day for eight days after infection and analyzed to verify the expression levels of virus genes, cytokines and *Nos2* transcripts by real-time PCR. Mice inoculated with uninfected C6/36 cell supernatants were used as controls. Quantitative PCR allowed the detection of viral transcripts in the mouse brains starting on the 2nd d.p.i. and revealed an increasing viral load up to 8 days after infection (Figure 2A). The transcript for cytokine genes *Ccl5*, *Cxcl10*, *Ccl3*, *Ifng*, *Ifnb*, and *Tnfa* in the mouse brain showed a similar profile, with increased expression levels between the 7th and 8th d.p.i. (Figure 2B-D, F-H). The increased transcript levels were statistically significant for all of the cytokines. The level of the *Nos2* transcript was higher on the 7th and 8th d.p.i. and was significant when compared to mice inoculated with uninfected C6/36 cell supernatants (Figure 2E). The presence of virus appears to correlate with increased *Nos2* and cytokine expression in the brain of WT mice between the 7th and 8th d.p.i. (Figure 2).

### *Nos2*^−/−^ mice infected with DENV-3 genotype I presented no clinical signs, although high viral titers were found in the brain at the 8th d.p.i.

**Figure 3:**
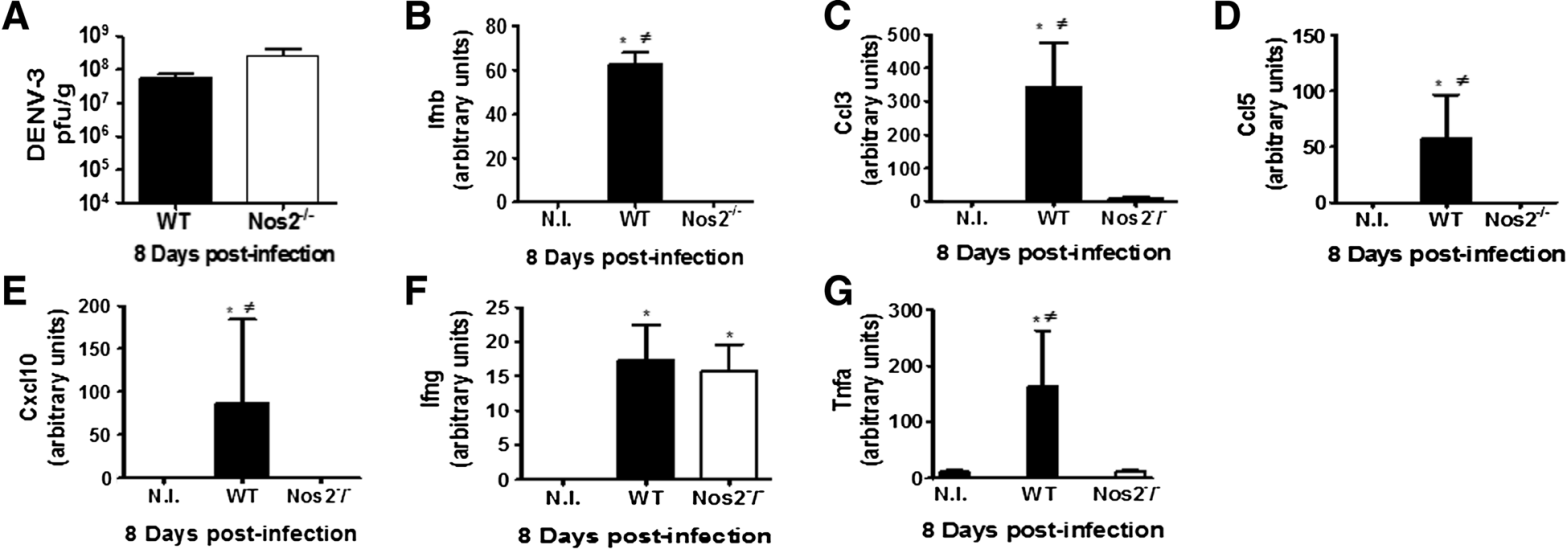
***Nos2***^**−/− **^**mice infected with DENV expressed lower levels of cytokines than WT mice, with exception of *Ifng *expression.** WT and *Nos2*^–/–^ mice were i.c. inoculated with 4 × 10^2^ pfu of DENV-3 (MG20) and the brains of 4 animals per group were collected at the 8th d.p.i. Mice inoculated with uninfected C6/36 cell supernatants were used as controls. RNA was extracted from the brains and reverse transcription and quantitative PCR were performed. **(A)** Infectious virus titers were determined as pfu/g of brains of the DENV-3 infected WT and *Nos2*^*−/−*^ mice at the 8th d.p.i.; **(B)***Ifnb*; **(C)***Ccl3*; **(D)***Ccl5*; **(E)***Cxcl10*; **(F)***Ifng*; **(G)***Tnfa*. * P < 0.05 when the infected mice were compared with the uninfected control mice (N.I.)*.≠* P < 0.05 when the infected WT mice were compared to the *Nos2*^*−/−*^ infected mice. The results are representative of two similar and independent experiments. Statistical analyses were performed using Kruskal-Wallis nonparametric tests and Dunn’s multiple comparison tests. Bars represent the SEM.

In contrast with the DENV-3-infected WT mice, 100% of the *Nos2*^−/−^ mice survived the infection, without exhibiting any clinical signs until the 30th d.p.i. To verify whether the virus replicates in the brains of *Nos2*^−/−^ mice, these mice were infected with 4 × 10^2^ pfu of DENV-3 genotype I and euthanized on the 8th d.p.i., which corresponds to the peak of viral replication in the WT mouse brains. Mice inoculated with uninfected C6/36 cell supernatants were used as controls. No differences in the viral load were detected in the brains of the WT and *Nos2*^−/−^ mice (Figure 3A). These results suggest a neuropathological role for NOS2 in DENV infection. The brains of infected mice that were euthanized on the 8th d.p.i. were analyzed by real-time PCR to verify whether there were different levels of cytokine expression in the infected WT and *Nos2*^–/–^ mice. The levels of pro-inflammatory cytokine expression increased consistently on the 8th d.p.i. in only the infected WT mice (Figure 3B-E, Figure 3G), with exception of *Ifng*, which increased in both the WT and in *Nos2*^*−/−*^ mice (Figure 3F).

### Fewer histopathological alterations were found in *Nos2*^−/−^ mice infected with DENV-3 genotype I (MG20)

**Figure 4:**
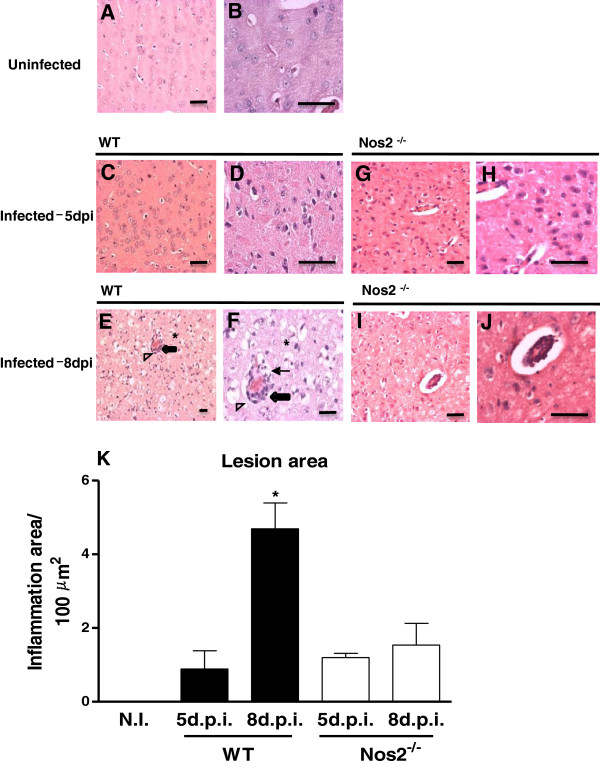
**Histopathological alterations that indicate neurovirulence were reduced in *Nos2***^**−/− **^**mice infected with DENV.** Mice were i.c. inoculated with 4 × 10^2^ pfu of DENV-3 (MG20). The brains of 4 animals per group were collected at day 5 or 8 post-infection (d.p.i.) and routinely processed for histopathological analysis. Mice inoculated with uninfected C6/36 cell supernatants were used as controls. Collected mice brains: **A** and **B**-Uninfected; **C** and **D**-DENV-3-infected WT at 5th d.p.i.; **E** and **F**- DENV-3-infected WT at 8th d.p.i.; **G** and **H**-DENV-3-infected *Nos2*^−/−^ at 5th d.p.i.; **I** and **J**-DENV-3-infected *Nos*^−/−^ at 8th d.p.i. Histopathological alterations were not observed in the brains of uninfected WT mice **(A** and **B)**. Note the endothelial response (thick arrows) and perivascular leukocyte cell migration (thin arrows) as well as the intense vacuolization of the brain tissue (arrowhead), suggesting neuronal degeneration, necrosis, and apoptosis (*) **(E** and **F)**. The images were captured at different magnifications, bar = 10 micrometers. **(K)** The area of the lesion was expressed as the inflammatory area per 100 μm^2^ of brain tissue. The results are representative of two similar and independent experiments. Data from the different groups were compared using a one-way analysis of variance (ANOVA) (GraphPad Prism software, San Diego, USA). *P < 0.05 when comparing the 8th d.p.i. in the WT mice with the other bars. N.I. = uninfected.

One-half of each infected brain from the mice that were euthanized on the 5th and 8th d.p.i. was formalin fixed for histopathological analysis. Brain sections from the mice that were inoculated with the uninfected C6/36 cell supernatant did not display any pathological changes at any time post-infection (Figure 4A and B). Histological analysis of the brains that were excised on the 5th d.p.i. from WT mice that were infected with DENV-3 genotype I revealed the preservation of nervous tissue with hyperemia, perivascular edema, and a sparse inflammatory infiltrate that adhered to the vessel walls (Figure 4C and D). Few inflammatory foci containing mononuclear cells could be detected in the meninges without involvement of the subjacent parenchyma. The histological analysis of brains from DENV-3-infected *Nos*2^−/−^ mice on the 5th d.p.i. did not differ from the brains of infected WT mice at the same time point (Figure 4G and H). At 8 d.p.i., the brains from DENV-3-infected WT mice exhibited diffuse and intense inflammation and edema of the nervous tissue, vessels and meninges (Figure 4E and F). Meningitis was characterized by several large inflammatory foci containing mononuclear and polymorphonuclear cells. In the parenchyma, intense vascular alterations, intracellular and pericellular edema, and degenerative changes in neurons and glial cells could be observed, although the inflammatory foci were rare and discrete. Vacuoles in the white and gray matter, irregular areas of necrosis, hemorrhage and recent thrombosis could be detected throughout the brain. At the same time point, the brains of DENV-3-infected *Nos2*^−/−^ mice did not present the histopathological alterations that were observed in the brains from infected WT mice (Figure 4I and J). The infected *Nos2*^−/−^ brain parenchyma exhibited few isolated necrotic or apoptotic areas, discrete perivascular inflammatory infiltrates, edema and vascular alterations.

### Increased levels of IFN gamma and NOS2 in the brains of infected mice are produced by CD4^+^ T cells and macrophages, respectively

**Figure 5:**
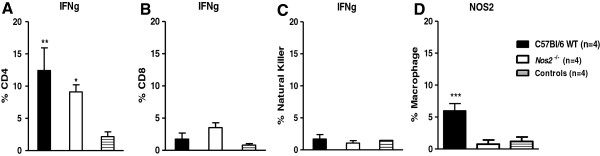
**DENV induces production of IFNg by CD4**^**+ **^**T cells and NOS2 by macrophages.** Mice were i.c. inoculated with 4 × 10^2^ pfu of DENV-3 (MG20) and the brains of 4 animals per group were collected on the 8th d.p.i. Mice inoculated with uninfected C6/36 cell supernatants were used as controls. The brains were treated with collagenase and Percoll (Sigma-Aldrich) and the percentage of IFNg- and NOS2-producing cells in the various cell populations was analyzed by flow cytometry: **(A)** IFNg in CD4^+^ T cells; **(B)** IFNg in CD8^+^ T cells; **(C)** IFNg in NK cells; **(D)** NOS2 in macrophages. *P < 0.05 and **P < 0.001 when comparing the infected mice to the uninfected mice **(A**-**C)***.***(D)** ***P < 0.0001 when comparing the infected versus uninfected WT and the infected *Nos2*^−/−^ mice versus the infected WT mice. Statistical analyses were performed using nonparametric two-way ANOVA tests and the Bonferroni post-hoc test.

To study the cell populations and the cytokine expression by the cells in the brains of infected mice, we used flow cytometric analysis. This analysis showed that there were more CD4^+^ T cells producing IFN gamma in the brains of infected WT or *Nos2*^−/−^ mice than in the brains of non-infected WT or *Nos2*^−/−^ mice. There was no difference in the number of CD4^+^ T cells producing IFN gamma in the brains of infected WT mice compared with the brains of infected *Nos2*^–/–^ mice (Figure 5A). Natural killer cells and CD8^+^ T cells were also present in the brain, but the percentage of these cells producing IFN gamma did not differ between the groups of mice (Figure 5B and C). The percentage of macrophages producing NOS2 in infected WT mouse brains was significantly higher than in the brains of non-infected WT mice (Figure 5D).

## Discussion

To date, some unique symptoms of dengue remain poorly explained. Thus, the previous guidelines for the classification of dengue diagnosis as DF, DHF and DSS were recently modified by the World Health Organization to dengue without warning signs, dengue with warning signs and severe dengue [12]. One of the criteria for severe dengue is involvement of the central nervous system (CNS) with impaired consciousness. Several reports exist in the literature of CNS involvement following dengue infection: 4% of 378 patients suspected of CNS infection [1], 9.5% of children with viral encephalitis [5], and 48% of 150 fatal cases including CNS involvement [4] were positive for dengue. In two other reports, 5% [6] and 13.5% of total dengue patients [7] were identified as having CNS involvement. However, there are serious difficulties in the study of dengue in animal models that include the immune response and immunopathology of dengue with CNS involvement, generally because unmodified DENV does not infect or cause symptoms in laboratory animals. Mouse models of dengue infection have been extensively explored, but none of these models has reproduced all of the clinical symptoms and manifestations of dengue infection that are observed in humans [30,31]. Cell culture passage and mouse adaptation allows the infection with a virus that mimics human disease in mice [29,32,33]. However, the alteration of virulence characteristics of the virus can impair the study of induced pathologies that depend on the viral strain, as is the case with neurovirulence. In the present study, we characterized the fatal neurovirulence of non-mouse-adapted DENV-3 (MG20) genotype I that was delivered by i.c. infection of C57BL/6 (WT) mice. *Nos2*^−/−^ mice were resistant to this virus. DENV-3 MG20 was originally isolated in 2004 from the serum of a fatal human dengue case in Belo Horizonte (MG, Brazil) that had CNS involvement [19]. Although neurological manifestations have been described in dengue, the disease’s true prevalence is unknown due to under-recognition of CNS presentations. Interestingly, the same clinical signs and mortality were observed in the DENV-3 MG20 infection in our study as were observed in DENV-1 Mochizuki-infected mice [22], a classical model of neurovirulence in mice.

In our model, immunocompetent mice had high levels of pro-inflammatory cytokine expression in the brain after infection with DENV-3 (MG20). The cytokine and viral gene transcripts exhibited a similar profile with increased expression levels peaking at the 7th to 8th d.p.i. and a concomitant increase in viral replication, paralysis, and neural tissue damage, culminating in death, demonstrating the involvement of the immune system in the pathogenesis after infection with DENV-3 (MG20). Molecules such as IFN gamma and TNF alpha appear to be markers for the different degrees of dengue disease or the dengue prognosis [14,34]. The DENV-3 (MG20)-infected *IFN-g*^−/−^ mice were susceptible to infection and exhibited 100% mortality, presenting the same clinical signs of infection as the infected WT mice. Microscopic examination of brains from mice that were infected with DENV-3 (MG20) revealed the presence of inflammatory infiltrates and edema in infected WT and *Nos2*^−/−^ mice on the 5th d.p.i. However, on the 8th d.p.i., the brains of the infected WT mice showed an intense endothelial response with perivascular polymorphonuclear cells, mononuclear leukocyte cell migration, meningitis and intense vacuolization that suggested neuronal degeneration, necrosis, and apoptosis. By contrast, the *Nos2*^−/−^ DENV-infected mice presented only mild histopathological changes in their brains at the 8th d.p.i. As the disease progressed, the infected WT animals appeared to be lethargic and displayed reduced motility. This factor may have resulted in reduced water intake and dehydration of the animals, contributing to the weight loss observed near the moribund stage, and consequently leading to death of the animals. We demonstrated intense vacuolization in the brains of infected mice, suggesting neuronal degeneration, necrosis, and apoptosis. Furthermore, increased *Nos2* expression at the 8th d.p.i. was observed, accompanied by a worsening of the clinical signs and a peak viral load in the brain. *Nos2* is responsible for the cytotoxic action of macrophages and neutrophils, and nitric oxide (NO) has been implicated in neurodegeneration and chronic inflammation in a mouse model of neurotoxicity. When NO production is associated with TNF alpha and IFN gamma in the brain, cerebral damage is caused, which induces an increase in the expression of the genes encoding pro-inflammatory molecules [35]. At optimal doses and when acting on cells of an organ able to regenerate quickly, NO has a protective and regulatory function; however, NO has toxic effects at higher concentrations [36] and when acting on cells of an organ which has difficulty regenerating such as the brain. Our results demonstrated that 100% of the *Nos2*^–/–^ mice survived infection with the DENV-3 (MG20) without exhibiting any clinical signs until the 15th d.p.i. Moreover, flow cytometric analysis revealed that CD4^+^ T cells, CD8^+^ T cells and macrophages were present in the brains of infected mice and that the CD4^+^ T cells were responsible for producing IFN gamma, which most likely induced the production of NOS2 by the WT mouse macrophages in response to DENV infection. Furthermore, the infected *Nos2*^−/−^ mice displayed no significant increase in cytokine transcripts in their brains with the exception of the *Ifng* transcript, which was also expressed at a higher level than in uninfected mice. These *Nos2*-deficient animals exhibited fewer histopathological alterations following infection, confirming the importance of NOS2 in the neuropathogenesis of neurovirulent DENV. By contrast, when mouse-adapted DENV-3 [32] was intraperitoneally injected into the *Nos2*^−/−^ mice, 100% mortality was observed, indicating that the virus isolate (DENV-3 genotype I or adapted DENV-3) and the route of inoculation (intracranial or intraperitoneal) are distinct variables responsible for these contrasting results. Indeed, each mouse model represents a unique tool to study the various patterns of pathologies caused by DENV.

Thus, i.c. infection of immunocompetent mice with a DENV isolate that causes CNS disease can be used as a tool to study the immune response, immunopathological manifestations, and neurological manifestations of dengue infection that are increasingly being reported in endemic dengue regions.

## Conclusions

In conclusion, DENV-3 genotype I (MG20) induces a virulent infection with CNS involvement and results in death of immunocompetent mice at a low viral dose and without requiring adaptation of the virus to mice. Nitric oxide synthase 2 is deleterious for the host in this experimental mouse infection model. Notably, our data indicate that this mouse model may be a useful tool for studying the immunopathology of dengue in the CNS.

## Competing interests

The authors declare that they have no competing interests.

## Authors’ contributions

KPRS, CMAL, RMEA, JASG, JGO, EGK, and MAC conceived of and designed the experiments. KPRS, EGS, ESOR, LBF, CMAL, RMEA, JASG, and GPF performed the experiments. KPRS, JGO, CMAL, RMEA, JASG, EGK, and MAC analyzed the data. RMEA, JASG, EGK, and MAC contributed reagents, materials, and analysis tools. KPRS, CMAL, RMEA, JASG, JGO, EGK, and MAC wrote the paper. All authors read and approved the final manuscript.
